# Gemcitabine, dexamethasone and cisplatin (GDP) is an effective and well-tolerated mobilization regimen for relapsed and refractory lymphoma: a single center experience

**DOI:** 10.3906/sag-2008-114

**Published:** 2021-04-30

**Authors:** Hikmettullah BATGİ, Semih BAŞCI1, Mehmet Sinan DAL1, Merih KIZIL ÇAKAR, Bahar UNCU ULU, Tuğçe Nur YİĞENOĞLU, Nurgül ÖZCAN, Ali KILINÇ, Alparslan MERDİN, Jale YILDIZ, Mehmet BAKIRTAŞ, Derya ŞAHİN, Tahir DARÇIN, Dicle İSKENDER, Nuran Ahu BAYSAL, Fevzi ALTUNTAŞ

**Affiliations:** 1 1Department of Hematology and Bone Marrow Transplantation Center, Ankara Dr. Abdurrahman Yurtaslan Oncology Training and Research Hospital, University of Health Sciences, Ankara Turkey; 2 Department of Clinical Biochemistry, Ankara Dr. Abdurrahman Yurtaslan Oncology Training and Research Hospital, University of Health Sciences, Ankara Turkey

**Keywords:** Gemcitabine, dexamethasone, cisplatin, stem cell mobilization, relapsed and refractory lymphoma

## Abstract

**Background/aim:**

Gemcitabine, dexamethasone and cisplatin (GDP) is a well-established salvage regimen for relapsed and refractory lymphomas. In this study, we aimed to share our experience with the patients who received GDP/R-GDP (rituximab-gemcitabine, dexamethasone and cisplatin) for stem cell mobilization.

**Materials and methods:**

Data of 69 relapsed and refractory Hodgkin lymphoma (HL) and Non-Hodgkin lymphoma (NHL) patients who received GDP/R-GDP as salvage chemotherapy in our center between July 2014 and January 2020 were retrospectively evaluated. After the evaluation of response, 52 patients had a chemosensitive disease and underwent mobilization with GDP/R-GDP plus G–CSF (granulocyte colony-stimulating factor). Collected CD34+ stem cells and related parameters were compared in terms of diagnosis of HL and NHL, early and late stage, patients who did not receive RT and those who received RT, and patients aged under 60 and over 60.

**Results:**

On the 15th day on average (range 11–20), a median number of 8.7 × 106 /kg (4.1–41.5) CD34+ stem cells were collected in 51 (98%) of our 52 chemosensitive patients and 1 (2%) patients failed to mobilize. We observed acceptable hematological and nonhematological toxicity. The targeted amount of 2 × 106 /kg CD34+ stem cells was attained by 98% (n: 51) patients, and all of them underwent autologous stem cell transplantation. Moreover, low toxicity profiles provide outpatient utilization option clinics with close follow-up and adequate supportive care.

**Conclusion:**

We suggest that GDP/R-GDP plus G-CSF can be used as an effective chemotherapy regimen for mobilizing CD34+ stem cells from peripheral blood in relapsed and refractory lymphoma patients due to low toxicity, effective tumor reduction, and successful stem cell mobilization. It can also be assumed that the GDP mobilization regimen may be more effective, especially in patients with early-stage disease and in HL patients.

## 1. Introduction

Autologous stem cell transplantation (ASCT), which is a highly therapeutic approach to the treatment of relapsed and refractory lymphoma, is extremely dependent on the mobilization and collection of hematopoietic stem cells (HSC) [1,2]. HSCs can be collected directly from the bone marrow or peripheral blood (PB) by apheresis. ASCTs are performed primarily with peripheral blood stem cells (PBSC). The release of HSCs to PB after granulocyte colony-stimulating factor (G-CSF) treatment and/or chemotherapy is known as mobilization. CD34+ cells do not exceed 0.05% of white blood cells (WBCs) under normal conditions in PB. After combining chemotherapy and G-CSF, the number of PBSC increases from 5 to 15 times [3–5].

The target quantity of HSC to be collected is dependent on the underlying disease (Non-Hodgkin lymphoma (NHL), Hodgkin lymphoma (HL), and the number of transplants. The minimum dose considered to be safe in case of ASCT is 2 × 106 CD34+ cells/kg per transplant; however, the aim of many centers is higher yields of 4–5 × 106 CD34+ cells/kg as it may allow faster neutrophil and platelet (PLT) recovery, reduced hospitalization, blood transfusions, and antibiotic therapy. The ideal dose required for successful transplantation was considered to be 5 × 106 CD34+ cells/kg [6–8]. The choice of a specific chemomobilization approach is based on the patient’s disease characteristics and local clinical practice guidelines. The applications that incorporate both the G-CSF and chemotherapy regimens were shown to mobilize more PBSCs than G-CSF alone [9,10].

The combination of G-CSF and chemotherapy is favored for stem cell mobilization and for tumor burden reduction and especially those who need to harvest a greater count of stem cells. It is an option to utilize mobilization not by splitting chemotherapy apart, however, through more precise, disease definite chemotherapy regimens such as; rituximab dexamethasone cytarabine cisplatin (R-DHAP) or rituximab, ifosfamide, carboplatin, and etoposide (R-ICE) for lymphoma patients [11]. After chemotherapy regimen employment, G-CSF daily dosage for mobilization was recommended as filgrastim 10 μg/kg and lenograstim 150 μg/m2. The G-CSF should be initiated following the fulfillment of chemotherapy instantly when leukocyte nadir is detected, and it should be continued till the ending of leukapheresis. Generally, it is recommended to begin G-CSF in 1–5 days following the completion of chemotherapy. Nonetheless, chemomobilization is not a panacea and has some detrimental aspects such as; therapy-associated toxicity, need for frequent hospitalization, harming bone marrow for forthcoming mobilizations and huge cost [11]. Also, it is known that repeated interventions for mobilization after failures constitute a burden for resource utilization and morbidity [12]. Considering all these factors together, determination of the most appropriate chemotherapy regimen for mobilization gains more importance [13].

The data regarding gemcitabine, dexamethasone, and cisplatin (GDP)/rituximab, gemcitabine, dexamethasone, and cisplatin (R-GDP) on stem cell mobilization are not widely investigated. This study is particularly designed to determine the results of GDP/R-GDP regimen plus G-CSF on mobilization as salvage therapy in patients with relapsed and refractory lymphoma. 

## 2. Materials and methods

Data of 69 relapsed and refractory HL and NHL patients who received GDP/R-GDP as salvage chemotherapy in our center between July 2014 and January 2020 were retrospectively evaluated. All the patients received GDP/R-GDP as salvage regimen (rituximab 375 mg/m2 on day 0, gemcitabine 1000 mg/m2 on days 1 and 8, cisplatin 75 mg/m2 on day 1, dexamethasone 40 mg/day on days 1, 2, 3 and 4: standard doses without dose modifications). Response assessment was based on imaging results from fluorodeoxyglucose–positron emission tomography-computed tomography (FDG/PET-CT) and computed tomography (CT) scans after treatments. The FDG/PET–CT and CT scans were evaluated by using Lugano criteria to assess FDG/PET–CT in lymphoma response criteria published in 2014 [14]. Fifty-two patients who received GDP/R-GDP had a chemosensitive disease. After GDP was given, it was the nadir for neutrophil to decrease and start to increase again, and G-CSF (2 × 5 g/kg/day) was started. Stem cell mobilization practice for lymphoma patients in our center was to start apheresis when the peripheral blood CD34+ count (PB CD34+) was > 10 cells/L, with a collection target of > 5 × 106 CD34+ cells/kg. Mobilization failure was defined as achieving a total CD34+ yield of < 2 × 106 cells/kg. Stem cell mobilization with GDP/R–GDP was compared in terms of diagnosis of HL and NHL, early and late stage, patients who did not receive RT and those who received RT, and patients under 60 and over 60 years of age.

### 2.1. Statistical analysis 

The SPSS version 21.0 (IBM Corporation, Armonk, NY, USA) was applied to analyses. The categorical variables were presented as frequency tables, and the numerical variables were presented as either mean ± standard deviations or median and minimum-maximum values, where appropriate. Distributions of continuous variables were assessed with graphics and Kolmogorov–Smirnov test. Mann–Whitney U test was implemented to compare the nonparametric continuous variables within the groups. A chi-square test was used to analyze apheresis count frequency between the groups. A P-value ≤ 0.05 was regarded as statistically significant.

## 3. Results 

GDP/R–GDP was given to 69 relapsed and refractory HL and NHL patients as salvage chemotherapy. Of the patients, 42 (60.9%) were males, and 27 (39.1%) were females. 38 (55%) patients had the diagnosis of HL, and 31 (45%) patients had NHL. The mean age of the patients was 43.9 ± 15.2 years. The demographic and clinical characteristics of the patients are summarized in Tables 1 and 2. After the evaluation of response to GDP or R-GDP regimen, a mobilization with G-CSF was performed for 52 patients who had a chemosensitive disease. On the 15th day, on average (range 11–20), ____ CD34+ stem cells were collected. The G-CSF mean was performed for 5 days (range 3–11). Peripheral CD34+ stem cell count before collection (on the day of collection) was between 11 and 467 cells?/μL, and median number of peak CD34+ stem cells in peripheral blood was 55 cells?/μL. The CD34+ stem cells were collected in 51 of our 52 chemosensitive patients (≈ 98%), and 1 (≈ 2%) patients failed to mobilize. In 51 patients, > 2 × 106 CD34+ stem cells/kg (median 8.68 × 106, range 4.06–41.50) were successfully collected. They were collected with one leukapheresis procedure in 34 patients, with two leukapheresis procedures in 15 patients, and with three leukapheresis procedures in 2 patients. The results of PBPCs collection are summarized in Table 3. 

**Table 1:** Demographic and clinical characteristics of the patients.

Diagnosis	HL (n: 38); NHL (n: 31)
Age	17–77 years (range) (mean age: 43.9)
Sex	Male (n:42); Female (n:27)
Disease status	Relapse (n:40); Refractory (n:29)
Radiotherapy	Yes (n:14); no. (n:55)
Previous number of chemotherapies	1 line (n: 60); 2 line (n: 7); 3 line (n: 1); 4line (n: 1)
GDP/R-GDP	GDP (n:42); R-GDP (n: 27)
Ann Arbor stage before GDP/R-GDP treatment	Stage 1 (n: 4); Stage 2 (n: 13); Stage 3(n: 16); Stage 4 (n: 36)
Bone marrow involvement	3/38 (8%); 8/31 (25.8%)
GDP/R-GDP number of cycles	2 (n: 36); 3 (n: 26); 4 (n: 7)
GDP/R-GDP treatment response	Chemorefractory disease n: 17;Chemosensitive disease n: 52
Stem cell mobilization with GDP/R-GDP	n: 52 (n: 51, 98% successful; n: 1, 2% unsuccessful)

**Table 2:** Clinical characteristics of patients.

Clinical characteristics	Number of patients (n)
Lymphoma type	69
Hodgkin’s lymphoma	38
Non-Hodgkin’s lymphoma	31
Diffuse large B-cell lymphoma	24
T-cell lymphoma	4
Mantle cell lymphoma	1
Follicular lymphoma	1
Marginal zone lymphoma	1
Previous chemotherapies	
Hodgkin’s lymphoma	
ABVD	27
ABVD + radiotherapy	11
Non-Hodgkin’s lymphoma	
R-CHOP	21
R-CHOP + radiotherapy	3
CHOP	4
R-EPOCH	2
CHOEP	1

**Table 3:** Results of peripheral blood stem cells collection.

Variable	All patients, n: 52
Median CD34+ cell count in peripheral blood (/μL) (range)	55.04 (11.07–467.18)
Median apheresis days (range)	15 (11–20)
Leukapheresis procedure count (n)	1 (n: 34); 2 (n: 15); 3 (n: 2)
Median total CD34+ cells collected (106/kg) (range)Out of target (< 2 × 106 CD34 + cells/kg) (%)	8.68 (4.06–41.50)1 (2)
Above minimum target (> 2×106 CD34 + cells/kg) (%)	51 (98)
Above optimal target (> 5×106 CD34 + cells/kg) (%)	48 (92)

Demographic and clinical characteristics of the patients with successful mobilization are summarized in Table 4. The mean age of the patients with successful mobilization (n: 51) was 44 ± 14.5 years. Twenty-nine (≈ 57%) were males and 22 (≈ 43%) were females. Twenty-four (≈ 47%) patients had the diagnosis of HL, and 27 (≈ 53%) patients had NHL. Of these, 22 were relapsed, 29 were refractory, and 15 had early stage and 36 had an advanced stage disease. The patient with unsuccessful mobilization was a 25-year-old female relapse Stage 3BX HL who had received one-line chemotherapy before and had a history of RT. Her response to GDP was a complete response. After nearly 3 weeks, CD34+ stem cells were collected with a G-CSF plus plerixafor.

**Table 4:** Demographic and clinical characteristics of successfully mobilized patients.

Variable	All patients, n: 51
Median age (range)	44 (18–77)
Sex	Male (n: 29); Female (n: 22)
Diagnosis	HL (n: 24); NHL (n: 27)
Disease status	Relapse (n: 22); Refractory (n: 29)
Stage 1–2, n	15
Stage 3–4, n	36
Patients undergoing radiotherapy, n	11
Median CD34 cell count in peripheral blood (cells?/μL)(range)	55.04 (11.07–467.18)
Previous line of chemotherapy	1 line (n: 45); 2 line (n: 4); 3 line (n: 1); 4 line (n: 1)

Blood parameters at the collection date are shown in Table 5. The PLT count was below 150 × 109 /L in 42 (82%) of 51 patients and below 100 × 109 /L in 34 (67%) of 51 patients. 2 patients (4%) had neutropenia (< 1.500 × 109 /L).

Grade 1–2 toxicity was approximately 5.9% (n: 3), which was ototoxicity, mucositis, and/or nephrotoxicity. Grade 3–4 toxicity was approximately 7.8% (n: 4), which was neutropenia (n: 2), febrile neutropenia (n: 1), infections requiring hospital admission (n: 2) and/or nephrotoxicity (n: 1).

**Table 5:** Blood parameters at harvest.

Variable	Median (range)
Leukocyte count (×109/L)	14 (2.44–53.6)
Hemoglobin level (g/dL)	11.2 (7.57–13.4)
Platelet count (×109/L)	62 (20–181)
Neutrophil count (×109/L)	8.7 (1.05–42.74)

Patients under 60 years of age had a higher number of CD34+ stem cells collected on day 1 than those over 60 years of age (P: 0.03). However, there was no difference in total CD34+ collected. The amount of premobilization PLT, apheresis day PB CD34+, CD34+ on the first day, and CD34+ total in HL patients were higher than in the NHL patients (P: 0.02, P: 0.002, P: 0.006, P: 0.03, respectively). In the early-stage patients, total CD34+ amount, and apheresis day PB CD34+ was found higher than in the late-stage patients (P: 0.02 and P: 0.04, respectively). As shown in Tables 6 and 7, when patients who received RT were compared with those who did not receive RT, no statistically significant difference was found in terms of WBC, PLT, and premobilization PB CD34+ stem cell counts, total number of collected CD34+ stem cells, number of CD34+ stem cells collected on the 1st day, and apheresis procedures.

**Table 6:** Relationship of mobilization and laboratory parameters with clinical variables.

Age				Diagnosis		
Median (min-max)	Aged < 60 (n: 39)	Aged ≥ 60 (n: 12)	P value	HL (n: 24)	NHL (n: 27)	P value
WBC	13.7(2.4–53.6)	14(6.6–34.1)	0.85	15.7(3.2–53.6)	10.1(2.4–46.2)	0.12
PLT	65.5(20–181)	52(20–107)	0.51	73(30–134)	46(20–181)	0.02*
PB CD34	73.6(11.1–467.2)	35.2(19.5–213)	0.12	119.3(19.5–467.2)	35.2(11.1–173.8)	0.002*
CD34 (1st)	6.5(2.3–41.5)	3.6(1.7–20)	0.03*	11.5(2.2-34.3)	4.3(1.7–41.5)	0.006*
CD34 (T)	9.5(4.1–41.5)	8.2(5.5–20)	0.31	12.4(4.7–34.3)	8.3(4.1–41.5)	0.03*
Apheresiscount	1(1–3)	2(1–3)	0.12	1(1–2)	2(1–3)	0.11

**Table 7:** Relationship of mobilization and laboratory parameters with clinical variables.

Stage				RT		
Median(min-max)	Early(n: 15)	Late(n: 36)	P value	RT(n:11)	Non-RT(n:40)	P value
WBC	17.6(3.2–53.6)	12.5(2.4–46.2)	0.19	14.7(6.6–34.9)	12.5(2.4–53.6)	0.33
PLT	74(24–134)	52(20–181)	0.10	97(36-123)	55(20-181)	0.15
PB CD34	106.7(33.1–337.6)	36.8(11.1–467.2)	0.02*	132.5(29.3–399.1)	50.68(11.1–467.2)	0.90
CD34 (1st)	10.6(3.3–20)	4.9(1.7–41.5)	0.09	6.5(2.0–13.3)	5.95(1.7–41.5)	0.98
CD34 (T)	12.5(4.1–20)	8.3(4.7–41.5)	0.04*	9.5(4.7–17.1)	9.14(4.1–41.5)	0.92
Apheresis count	1(1–2)	1(1–3)	0.33	1(1–2)	1(1–3)	0.71

## 4. Discussion

Currently, the number of 2 **×** 106 CD34+ cells/kg is generally considered to be the minimum stem cell count needed for a successful ASCT. Ideally, the optimum value is generally considered to be > 5 **×** 106 CD34+ cells/kg, and the sum of collected stem cells below < 2 **×** 106 CD34+ cells/kg is regarded as mobilization failure [6,7,8,15].

Various chemotherapeutic agents are used in conjunction with G-CSF for stem cell mobilization in ASCT. Chemotherapeutic agents should be both effective against the underlying disease and should also facilitate stem cell mobilization; thus, both cytoreduction and mobilization should be provided together. This is the reason why single agents such as cyclophosphamide, etoposide, cytarabine, etc. are used along with G-CSF for both pretransplant cytoreduction and stem cell mobilization; therefore, combined regimens such as GDP, cisplatin, cytosine arabinoside and dexamethasone (DHAP), doxorubicin, methylprednisolone, high-dose cytarabine and cisplatin (ASHAP), Vinorelbine, gemcitabine, procarbazine and prednisone (ViGePP) and ifosfamide, carboplatin, and etoposide phosphate (ICE) have been used as stem cell mobilizing regimens in hematology units [16–18]. By using salvage chemotherapy in patients with relapsed or refractory HL, failure of 3%, 18%, and 14% mobilization rates were reported for GDP, carmustine cytarabine etoposide melphalan (Mini-BEAM), and ICE, respectively [16,17].

Bozdağ et al. investigated the effect of chemotherapy regimens on mobilization in lymphoma patients [18]. Patients were given chemotherapy protocols such as cyclophosphamide (n: 15), ASHAP (n: 11), and ViGePP (n: 12) [18]. Although no difference was reported between the groups concerning the number of stem cells collected (P: 0.58), mobilization failure was 33% in the cyclophosphamide group (n: 5/15), 9% in the ASHAP group (n: 1/11) and 8% in the ViGePP group (n: 1/12) [18]. 

Berber et al. evaluated the effectiveness of the DHAP regimen plus filgrastim for mobilization of stem cells in relapsed and/or refractory lymphoma patients [19]. Stem cells from 32 patients (94%) were collected on the 11th day on average and the median CD34+ cell count collected was 9.7 × 106 /kg (range 3.8–41.6) [19]. Mobilization failure in salvage treatments was reported as 10% in diffuse large B-cell lymphoma (DLBCL) (n: 197) patients given R-ICE, and it was 8% in DLBCL (n: 191) patients given R-DHAP [20]. Moccia et al. provided GDP salvage treatment to 235 relapsed and refractory HL and NHL patients in their study [21]. Autologous stem cell transplantation was applied to 126 patients (69 HL and 57 DLBCL) in total [21]. In addition, Moccia AA et al. also reported GDP as an effective out-patient salvage regimen for relapsed and refractory DLBCL and HL. However, in the study, the effectiveness of GDP on PBSC mobilization has not been adequately evaluated [21].

In the current study, we evaluated the efficacy of the GDP/R-GDP regimen plus G-CSF to mobilize PBSCs in relapsed and refractory lymphoma patients. Successful mobilization was achieved in 51 of chemosensitive patients and approximately 98% of patients had stem cells collected over 2 × 106 cells?/kg. Our mobilization failure was nearly 2%, and our mobilization failure seemed to be lower when compared to the reports of Mini-BEAM, ICE, cyclophosphamide, ASHAP, ViGePP, R-ICE, and R-DHAP regimens usage reported previously [15–18]. Besides, our study suggests that GDP mobilization regimen may be more effective in HL patients in comparison to NHL patients in terms of premobilization PLT levels, PB CD34+ stem cell counts, first-day collected stem cell amount of the mobilization, and the total number of CD34+ stem cells collected as shown in Tables 6 and 7.

Plerixafor could be added to G-CSF at a dose of 24 µg/kg when there is a possibility of inadequate mobilization (defined as PB CD34+ stem cell number < 10 cells/L on the first apheresis day planned or target CD34+ stem cell yield on the first day of apheresis < 50%) [23,24]. Tang C et al. used 4% and 18% plerixafor in regimens (CE (cyclophosphamide/etoposide) + G-CSF and GDP + G-CSF), respectively [24]. Besides, they reported the mobilization failure as 1.2% [24]. In our study, mobilization failure was 2% and only 1 patient used G-CSF plus plerixafor. Eventually, GDP regimen seemed not to need very high rates of plerixafor usage.

Patient and disease-related factors predicting mobilization failure are being over 60 years of age, having an underlying advanced disease, having previously received more than one-line chemotherapy, and having low CD34+ cells in peripheral blood before apheresis. However, the low PLT count before mobilization and previous treatments, including fludarabine, melphalan, or lenalidomide are controversial factors in terms of mobilization failure. It is generally accepted that the most influential predictive factor for mobilization failure is the number of CD34+ cells in preapheresis PB [6].

From a total of 145 patients, 52% of whom were diagnosed with lymphoma, participated in a study conducted by Demiriz et al. [25]. The patients were divided into two groups according to successful and unsuccessful mobilization and the groups were compared in terms of the parameters affecting the mobilization success [25]. Among the factors of age, platelet count, LDH, ferritin, CRP, LDL, and triglyceride levels, it was only high platelet count that was shown to be effective in mobilization success in their study (P < 0.05) [25]. On the other hand, due to the high platelet count before mobilization, the number of stem cells collected in HL patients was found to be higher than in NHL patients in this study and it would be an indicator of bone marrow reserve.

Dogu MH et al. showed that age, the number of chemotherapy cycles taken before mobilization, and radiation therapy had no significant effect on the number of final CD34+ stem cell yield (P: 0.492, 0.746, and 0.078, respectively) [26]. On the contrary, in our study, the amounts of CD34+ stem cells collected on the 1st day in patients under 60 years of age and older than that were different; however, total amounts of collected CD34+ stem cells were similar. However, there was no difference in terms of the amount of collected total CD34+ between the patients who received RT and those who did not. In addition, when early stage patients were compared with late stage patients, the total number of collected CD34+ stem cells was found to be significantly higher in the early stage patients.

Tang C et al. examined the efficacy and safety of PBSC mobilization following CE + G-CSF versus GDP + G-CSF [24]. Patients mobilized with CE + G-CSF required fewer days of leukapheresis (median 1 vs. 2 days; P: 0.001) and achieved a higher total CD34+ stem cell yield than patients mobilized with GDP + G-CSF (8.5 × 106 vs. 7.1 × 106 CD34+ cells/kg; P: 0.001) [24]. Frequencies of febrile neutropenia and rates of CD34+ stem cell collection ≥ 5 × 106 CD34+ cells/kg were similar [24]. Furthermore, in our study, GDP/R-GDP regimens provided a median number of 8.68 × 106 cells?/kg of CD34+ stem cells (range 4.06–41.50) PBSCs. Total CD34+ stem cell yield was collected by one leukapheresis procedure in 34 (≈ 66.7%) patients, 2 leukapheresis procedures in 15 patients (≈ 29.4%), and 3 leukapheresis procedures in 2 (≈ 3.9%) patients. 

In conclusion, we observed acceptable hematological and nonhematological toxicities with R-GDP/GDP salvage chemotherapies used in relapsed and refractory lymphoma patients. We also showed high rates of successful stem cell mobilization in relapsed and refractory lymphoma patients receiving GDP/R-GDP salvage chemotherapies. Therefore, GDP/R-GDP chemotherapy regimens should also be kept in mind as an alternative for salvage chemotherapy followed by peripheral stem cell mobilization in patients with relapsed and refractory lymphoma. It can also be assumed that a GDP mobilization regimen may be more effective, especially in patients with early-stage disease and also HL patients.

## Ethical approval

Local ethics committee approval was obtained.
